# Does the Chemotherapy Backbone Impact on the Efficacy of Targeted Agents in Metastatic Colorectal Cancer? A Systematic Review and Meta-Analysis of the Literature

**DOI:** 10.1371/journal.pone.0135599

**Published:** 2015-08-14

**Authors:** David L. Chan, Nick Pavlakis, Jeremy Shapiro, Timothy J. Price, Christos S. Karapetis, Niall C. Tebbutt, Eva Segelov

**Affiliations:** 1 Royal North Shore Hospital, Northern Clinical School, University of Sydney, St Leonards, New South Wales, Australia; 2 Department of Medical Oncology, Monash University, Victoria, Australia; 3 The Queen Elizabeth Hospital and University of Adelaide, South Australia, Australia; 4 Flinders University and Flinders Medical Centre, Flinders Centre for Innovation in Cancer, Bedford Park, South Australia, Australia; 5 Austin Health, Victoria, Australia; 6 St Vincent’s Clinical School, University of New South Wales, NSW, Australia; Catalan Institute of Oncology, SPAIN

## Abstract

**Importance:**

The EGFR inhibitors (EGFR-I) cetuximab and panitumumab and the angiogenesis inhibitors (AIs) bevacizumab and aflibercept have demonstrated varying efficacy in mCRC.

**Objective:**

To document the overall impact of specific chemotherapy regimens on the efficacy of targeted agents in treating patients with mCRC. Data sources: MEDLINE, EMBASE and Cochrane databases were searched to 2014, supplemented by hand-searching ASCO/ESMO conference abstracts.

**Study Selection:**

Published RCTs of patients with histologically confirmed mCRC were included if they investigated either 1) chemotherapy with or without a biological agent or 2) different chemotherapy regimens with the same biological agent. EGFR-I trials were restricted to *KRAS* exon 2 wild-type (WT) populations.

**Data Extraction and Synthesis:**

Data were independently abstracted by two authors and trial quality assessed according to Cochrane criteria. The primary outcome was overall survival with secondary endpoints progression free survival (PFS), overall response rate (ORR) and toxicity.

**Results:**

EGFR-I added to irinotecan-based chemotherapy modestly improved OS with HR 0.90 (95% CI 0.81–1.00, p = 0.04), but more so PFS with HR 0.77 (95% CI 0.69–0.86, p<0.00001). No benefit was evident for EGFR-I added to oxaliplatin-based chemotherapy (OS HR 0.97 (95% CI 0.87–1.09) and PFS HR 0.92 (95% CI 0.83–1.02)). Significant oxaliplatin-irinotecan subgroup interactions were present for PFS with I^2^ = 82%, p = 0.02. Further analyses of oxaliplatin+EGFR-I trials showed greater efficacy with infusional 5FU regimens (PFS HR 0.82, 95% CI 0.72–0.94) compared to capecitabine (HR 1.09; 95% CI 0.91–1.30) and bolus 5FU (HR 1.07; 95% CI 0.79–1.45); subgroup interaction was present with I^2^ = 72%, p = 0.03. The oxaliplatin-irinotecan interaction was not evident for infusional 5FU regimens. For AIs, OS benefit was observed with both oxaliplatin-based (HR 0.83) and irinotecan-based (HR 0.77) regimens without significant subgroup interactions. Oxaliplatin+AI trials showed no subgroup interactions by type of FP, whilst an interaction was present for irinotecan+AI trials although aflibercept was only used with infusional FP (I^2^ = 89.7%, p = 0.002).

**Conclusion and Relevance:**

The addition of EGFR-I to irinotecan-based chemotherapy has consistent efficacy, regardless of FP regimen, whereas EGFR-I and oxaliplatin-based regimens were most active with infusional 5FU. No such differential activity was observed with the varying chemotherapy schedules when combined with AIs.

## Introduction

Biologic agents have been extensively investigated in metastatic colorectal cancer (mCRC), both in combination with chemotherapy[1–21] and as monotherapy.[22, 23] Inconsistent results from combination therapy trials have been postulated to relate to interaction with chemotherapy partners, both with regard epidermal growth factor receptor inhibitors (EGFR-I) [24],[25] and anti-angiogenesis inhibitors (AIs) [26]. We undertook systematic review and meta-analysis to evaluate the overall effect of chemotherapy partner choice when combined with biological agents used in routine clinical care of patients with mCRC, i.e. the EGFR-I cetuximab [2, 3, 12, 18–20, 27] and panitumumab[16, 21], as well as the AIs bevacizumab[1, 4–9, 11, 13, 15, 17, 28] and aflibercept[14, 29]. The effect of type of FP, whether oral (capecitabine), infusional or bolus was also explored.

## Methods

### Search strategy

Publication databases (MEDLINE, EMBASE and Cochrane Trials Registry—to 31 October 2014) were searched (S1 Methods) and proceedings of major conferences (ASCO, ASCO GI, ESMO to January 2015) were handsearched. This study was not prospectively registered with a central registry. Unpublished data was sought from authors.

### Eligibility criteria

Published randomized controlled trials of any language or year were eligible for inclusion. Participants included were patients with metastatic (or advanced, unresectable) colorectal cancer.

Interventions studied were EGFR-I or AIs. EGFR-I trials were restricted to *KRAS* exon 2 wild-type (WT) populations. Eligible comparisons were 1) chemotherapy with biological agent versus chemotherapy alone or 2) different chemotherapy regimens with the same biological agent.

Search results were evaluated independently by two authors (DC, NP/ES), with disagreements in eligibility resolved by consensus after reference to the full text of the article. Data was extracted into piloted forms and double-checked by another author to ensure accuracy.

### Endpoints

The primary endpoint was overall survival (OS); secondary endpoints were progression free survival (PFS), overall response rate (ORR) and toxicity. Quality of life (QoL) data was extracted where available.

Other data extracted included PICOS, the quality/description of randomization, and any relevant funding sources. Risk of bias was performed at the study level, using the Cochrane risk of bias tool, with summary risk of bias as per Cochrane recommendations.

The principal summary measures were hazard ratio (HR) for OS/PFS and odds ratios for ORR and toxicity. Meta-analysis was carried out using the generic inverse variant method, with fixed-effects analysis and calculation of HR/OR as applicable with 95% confidence intervals (CI).

Trials were characterized by type of biologic and chemotherapy backbone. The two groups of biological therapy investigated were:

1. EGFR-I: with oxaliplatin (ox) backbone vs with irinotecan (iri) backbone.
2. AIs: with ox backbone vs with iri backbone vs FP alone.

Subgroup analysis was performed by type of FP: capecitabine, infusional or bolus. The mIFL regimen was considered in the bolus group.

Given the increasing literature on the improved efficacy of EGFR-I in extended RAS settings, we performed additional analysis for OS in trials that reported this outcome in extended RAS wildtype populations.

Heterogeneity was explored when I^2^>50% and p<0.10. Sensitivity analyses and funnel plots were undertaken to investigate possible bias.

## Results

### Study selection

**Fig 1:**
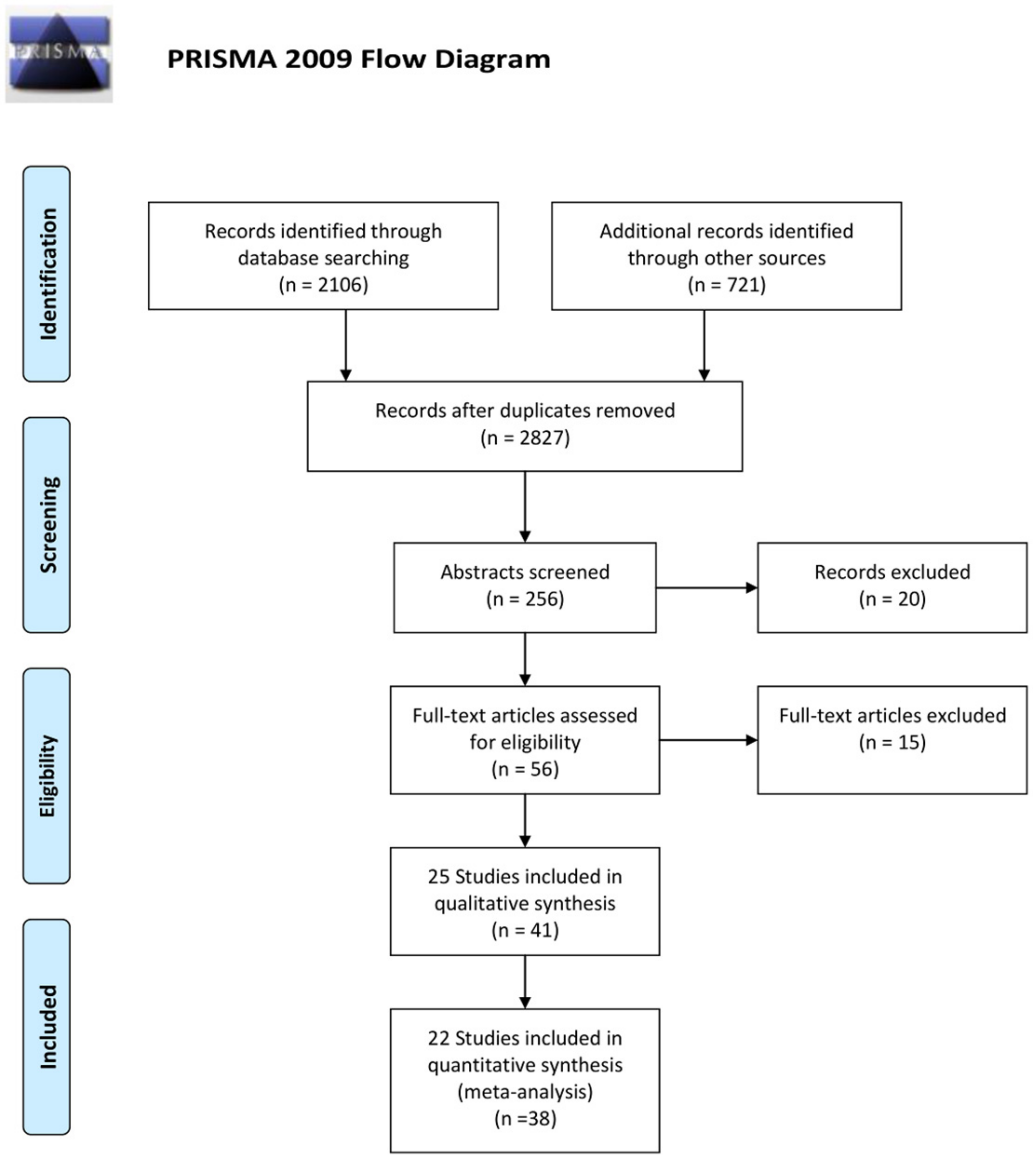
PRISMA flow diagram. *From*: Moher D, Liberati A, Tetzlaff J, Altman DG, The PRISMA Group (2009). *P*referred *R*eporting *I*tems for *S*ystematic Reviews and *M*eta-*A*nalysis: The PRISMA Statment. PLoS Med 6(6): e1000097. doi:10.1371/journal.pmed1000097 For more information, visit www.prisma-statement.org.

The literature search identified 256 potentially eligible citations from 2827 search results. Thirty-nine papers representing 23 studies comprising 10478 patients were eligible for inclusion (Table 1, Fig 1). The EPIC trial [30] was excluded, as analysis by KRAS exon 2 status was available for only 300/1298 patients, with incomplete OS and PFS data. Upon clarification with the lead author, we confirmed that insufficient data was currently available to enable meta-analysis and that there were no active plans for this analysis to be undertaken in the future. The PEAK trial, comparing FOLFOX + cetuximab to FOLFOX + bevacizumab in the first-line setting, was not included in quantitative analysis because it did not investigate the activity of either cetuximab or bevacizumab alone in addition to chemotherapy but rather compared its effects. Furthermore, both arms received the same chemotherapy backbone, meaning that it does not address the research question posed. The other studies comparing anti-EGFR to anti-angiogenesis agents with the same backbone (SPIRITT, FIRE-3) are excluded for the same reason.

**Table 1:** List of included trials.

**Studies evaluating the addition of a biologic agent to chemotherapy (19 trials, N = 9595)**							
Name	Author	Line	Experimental arm	Comparator arm	Number of pts	Risk of bias	Phase
**EGFR Inhibitors (9 trials, N = 3492)**							
**Oxaliplatin backbone (N = 2061)**							
OPUS	Bokemeyer (2009)	1^st^	FOLFOX + Cet	FOLFOX	134	L	III
PRIME	Douillard (2010)	1^st^	FOLFOX + Pan	FOLFOX	656	L	III
COIN	Maughan (2011)	1^st^	FOLFOX/CAPOX + Cet	FOLFOX/CAPOX	729 (243 FOLFOX, 472 CAPOX, 14 did not start)	L	III
NORDIC VII	Tveit (2012)	1^st^	FLOX + Cet, Intermittent FLOX + Cet	FLOX	303	L	III
New EPOC	Primrose (2013)	1^st^	Perioperative FOLFOX/CAPOX + Cet	FOLFOX/CAPOX	182 FOLFOX, 57 CAPOX	L	III
**Irinotecan backbone (N = 1431)**							
CRYSTAL	Van Cutsem (2009)	1^st^	FOLFIRI + Cet	FOLFIRI	348	L	III
Study 181	Peeters (2010)	2^nd^	FOLFIRI + Pan	FOLFIRI	597	L	III
PICCOLO	Seymour (2013)	2^nd^	Irinotecan + Pan	Irinotecan	460	L	III
New EPOC	Primrose (2013)	1^st^	Perioperative FOLFIRI + Cet	FOLFIRI	26 FOLFIRI	L	III
**Anti-VEGF agents (10 trials, n = 6103)**							
**Oxaliplatin backbone (n = 2454)**							
NO16966	Saltz (2008)	1^st^	FOLFOX/XELOX + Bev	FOLFOX/XELOX	700 FOLFOX, 700 XELOX	L	III
E3200	Giantonio (2007)	2^nd^	FOLFOX + Bev	FOLFOX	577	L	III
TML	Arnold (2012)	2^nd^	Multiple chemotherapies + Bev	Multiple Chemotherapies	477 oxali	L	III
ITACA	Passardi (2015)	1st	FOLFOX/FOLFIRI+Bev	FOLFOX/FOLFIRI	221 oxali	L	III
**Irinotecan backbone (n = 2585)**							
ARTIST	Guan (2011)	1^st^	mIFL + Bev	mIFL	203	L	III
AVF2107g	Hurwitz (2004)	1^st^	IFL + Bev	IFL	813	U	III
VELOUR	Van Cutsem (2012)	2^nd^	FOLFIRI + aflibercept	FOLFIRI	1226	L	III
TML	Arnold (2012)	2^nd^	Multiple chemotherapies + Bev	Multiple chemotherapies	343 iri	L	III
ITACA	Passardi (2015)	1st	FOLFOX/FOLFIRI+Bev	FOLFOX/FOLFIRI	145 iri	L	III
**Fluoropyrimidine alone (n = 1064)**							
AGITG MAX	Tebbutt (2010)	1^st^	XB, (XB+Mitomycin C)	Cape	471	L	III
AVF0780g	Kabbinavar (2003)	1^st^	FUFA + Bev 5mg/kg, FUFA + Bev 10mg/kg	FUFA	104	L	II
AVF2192g	Kabbinavar (2005)	1^st^	FUFA + Bev	FUFA	209	L	II
AVEX	Cunningham (2013)	1^st^	XB	Cape	280	L	III
**Studies evaluating different chemotherapy regimens added to the same biological agent (4 trials, N = 517)**							
Name	Author	Line	Experimental arm	Comparator arm	Number of pts	Risk of bias	Phase
KRK0104	Moosmann (2011)	1^st^	XELIRI + Cet	XELOX + Cet	89	L	II
CECOG	Ocvirk (2010)	1^st^	FOLFIRI + Cet	FOLFOX + Cet	62	U	II
CELIM	Folprecht (2010)	1^st^	FOLFIRI + Cet	FOLFOX + Cet	111	L	II
	Schmeigel (2013)	1^st^	CAPIRI + Bev	CAPOX + Bev	255	L	II

Abbreviations: Cet—Cetuximab, Pan—Panitumumab, Bev—Bevacizumab, XB—Capecitabine + Bevacizumab, Cape—Capecitabine

The Ye study [20] (investigating the addition of cetuximab to FOLFOX/FOLFIRI) met the set requirements, but was excluded from analysis as no results were available separately for the FOLFOX and FOLFIRI arms. PACCE and CAIRO2 were excluded given that both arms contained at least one biological agent (bevacizumab).

### Risk of Bias

The overall quality of the studies was good (Table 1). Funnel plots for PFS show possible publication bias with AIs (S2 Fig).

#### 1. The effect of chemotherapy partner on efficacy of EGFR-I

**Fig 2:**
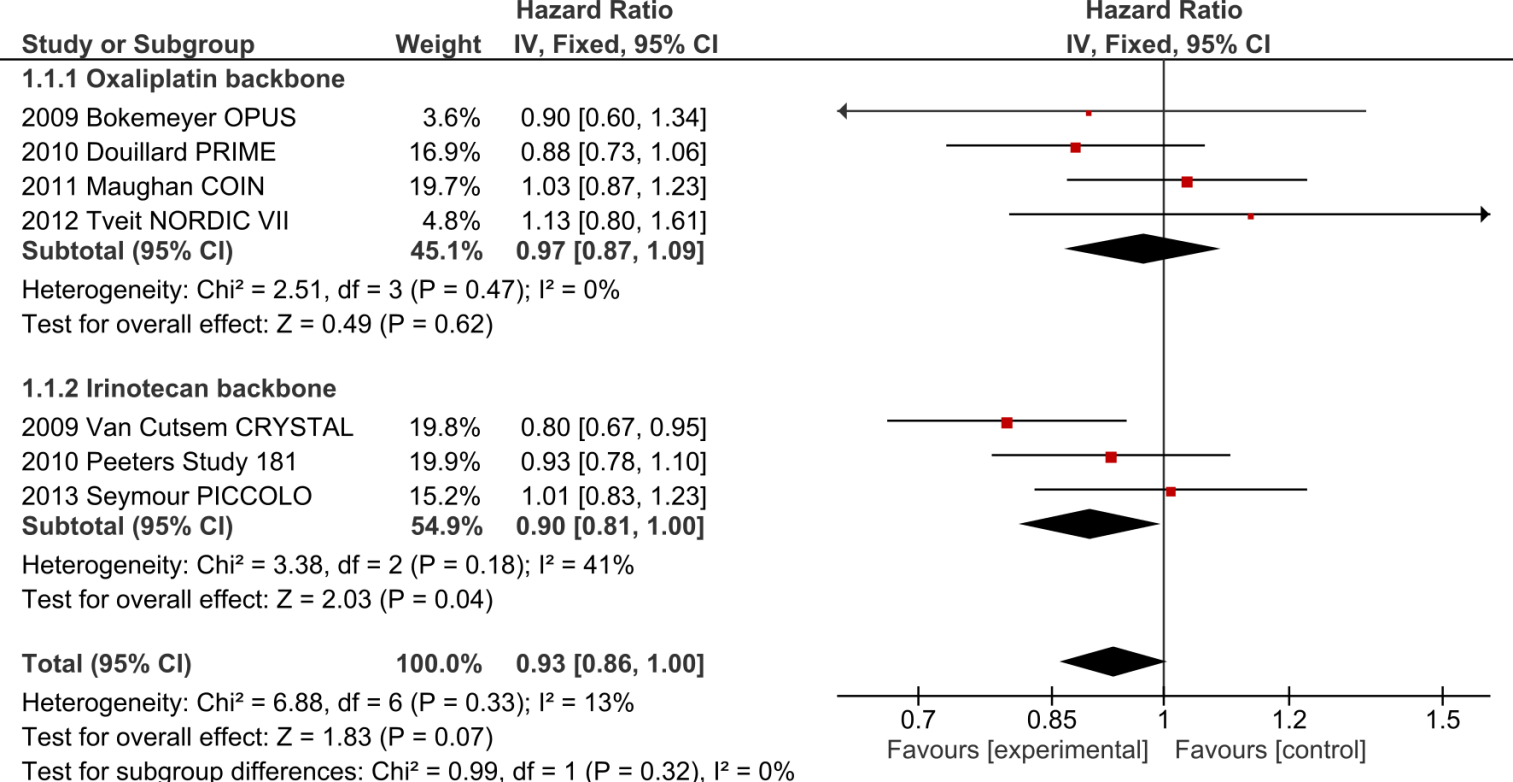
OS outcomes for EGFR-I by chemotherapy backbone.

**Fig 3:**
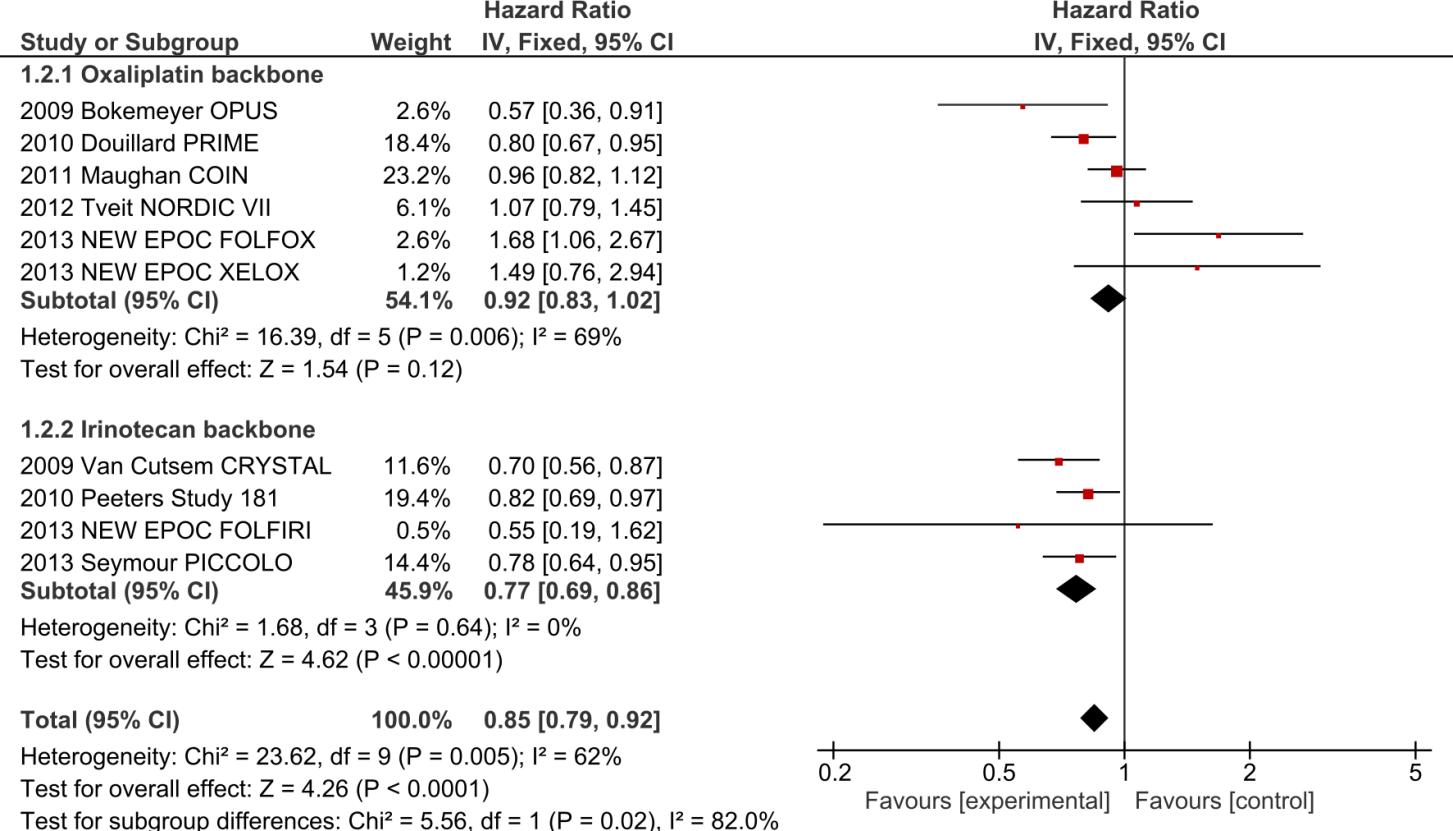
PFS outcomes for EGFR-I by chemotherapy backbone.

**1.1 Oxaliplatin backbone + EGFR-I**. Five studies (COIN[12], OPUS[2], PRIME[21], NEW EPOC[27] and NORDIC VII[18]), involving 2061 patients, investigated the addition of EGFR-I to oxaliplatin-based chemotherapy. The addition of EGFR-I did not improve OS (HR 0.97, 95% CI 0.87–1.09, p = 0.62, Fig 2) nor PFS (HR 0.92, 95% CI 0.83–1.02, p = 0.13, Fig 3). Overall Response Rate (ORR) was improved by 7.5% with odds ratio (OR) 1.36 (95% CI 1.12–1.64, p = 0.002). Significant heterogeneity was present in the PFS analysis (I^2^ = 69%, p = 0.006), possibly due to differences in the clinical settings and the use of different fluoropyrimidine backbone across the studies.

**Fig 4:**
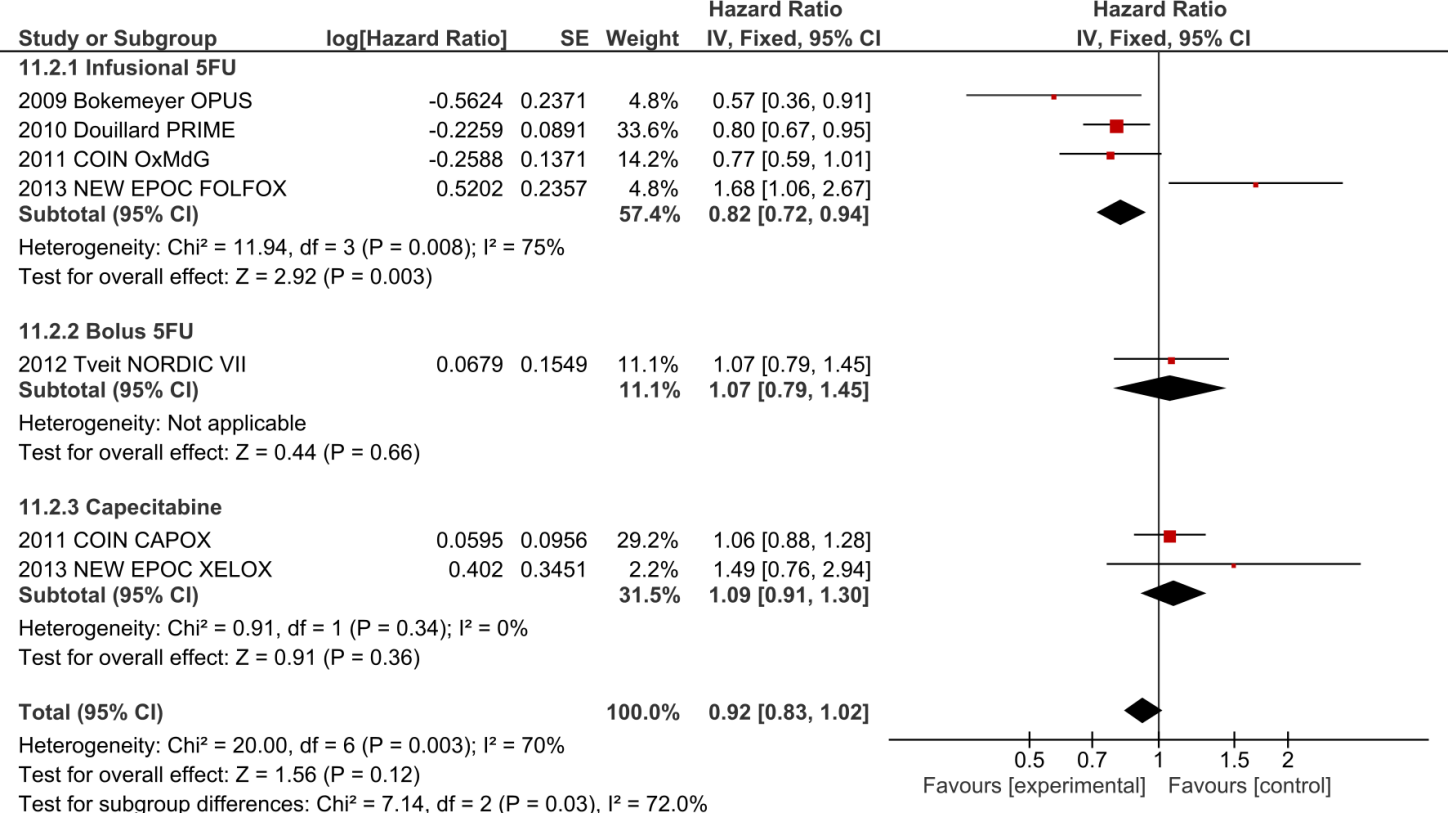
Fluoropyrimidine subgroup analysis for PFS–combining EGFR-I with oxaliplatin-based chemotherapy.

1.1.1. Impact of FP type on Oxaliplatin + EGFR-I: Analysis by type of FP was performed in the above trials. No significant interaction was present for OS (S3 Fig) but significant differences were noted for PFS (I^2^ = 72%, p = 0.03, Fig 4), with the infusional 5FU group demonstrating a PFS benefit (HR 0.82 (95% CI 0.72–0.94)) in contrast to the capecitabine (HR 1.09, 95% CI 0.91–1.30) and bolus FP (HR 1.07, 95% CI 0.79–1.45) groups. Only two studies evaluating capecitabine (n = 529 patients) were included in the PFS analysis by FP, but only one study (COIN) was included in the OS analysis, as data from the NEW EPOC Study for OS was not available to include.

**1.2 Irinotecan backbone + EGFR-I**. Four trials (CRYSTAL[19], Study 181[22], PICCOLO [16] and New EPOC [27]), involving 1431 patients, investigated the addition of EGFR-I to irinotecan-based chemotherapy. Addition of EGFR-I improved OS (HR 0.90, 95% CI 0.81–1.00, p = 0.01, Fig 2) as well as PFS (HR 0.77, 95% CI 0.69–0.86, p<0.00001, Fig 3). ORR was improved by +21.3% with OR 3.09 (95% CI 2.47–3.86, p<0.00001). Significant heterogeneity was present in the ORR analysis (I^2^ = 85%, p<0.0001) but ORR was still improved in random-effects analysis (OR 3.53, 95% CI 1.88–6.65). Analysis by FP type was not performed as trials utilized only FOLFIRI or single agent irinotecan backbones.

**1.3 Interaction between oxaliplatin and irinotecan with EGFR-I**. In comparing trials combining EGFR-I with ox to those combining EGFR-I with iri, significant interaction was present for PFS (I^2^ = 71.2%, p = 0.06, Fig 2) and ORR (I^2^ = 96.7%, p<0.00001) but not OS (I^2^ = 0%, p = 0.32). When the analysis was restricted to those utilizing infusional FP regimens (i.e. FOLFOX and FOLFIRI), interaction for PFS was no longer present (PFS I^2^ = 0%, p = 0.49, S4 Fig) although the ORR interaction persisted (I^2^ = 90.5%, p = 0.001), suggesting that choice of FP may be responsible for the interaction between the oxaliplatin-containing v irinotecan-containing regimens. To highlight this point, one can see that the pooled HR for PFS with all oxaliplatin containing regimens is 0.92 (95% CI 0.83–1.02) as compared with irinotecan containing regimens (HR 0.77; 95% CI 0.69–0.86) (Fig 2). When only infusional 5FU regimens are considered (S4 Fig), the pooled PFS HR for oxaliplatin containing regimens is 0.82 (95% CI 0.72–0.94) as compared with irinotecan containing regimens (HR 0.77; 95% CI 0.67–0.88). Thus greater PFS efficacy and confidence is observed with infusional 5-FU regimens and oxaliplatin than with bolus or capecitabine based oxaliplatin combinations.

**Fig 5:**
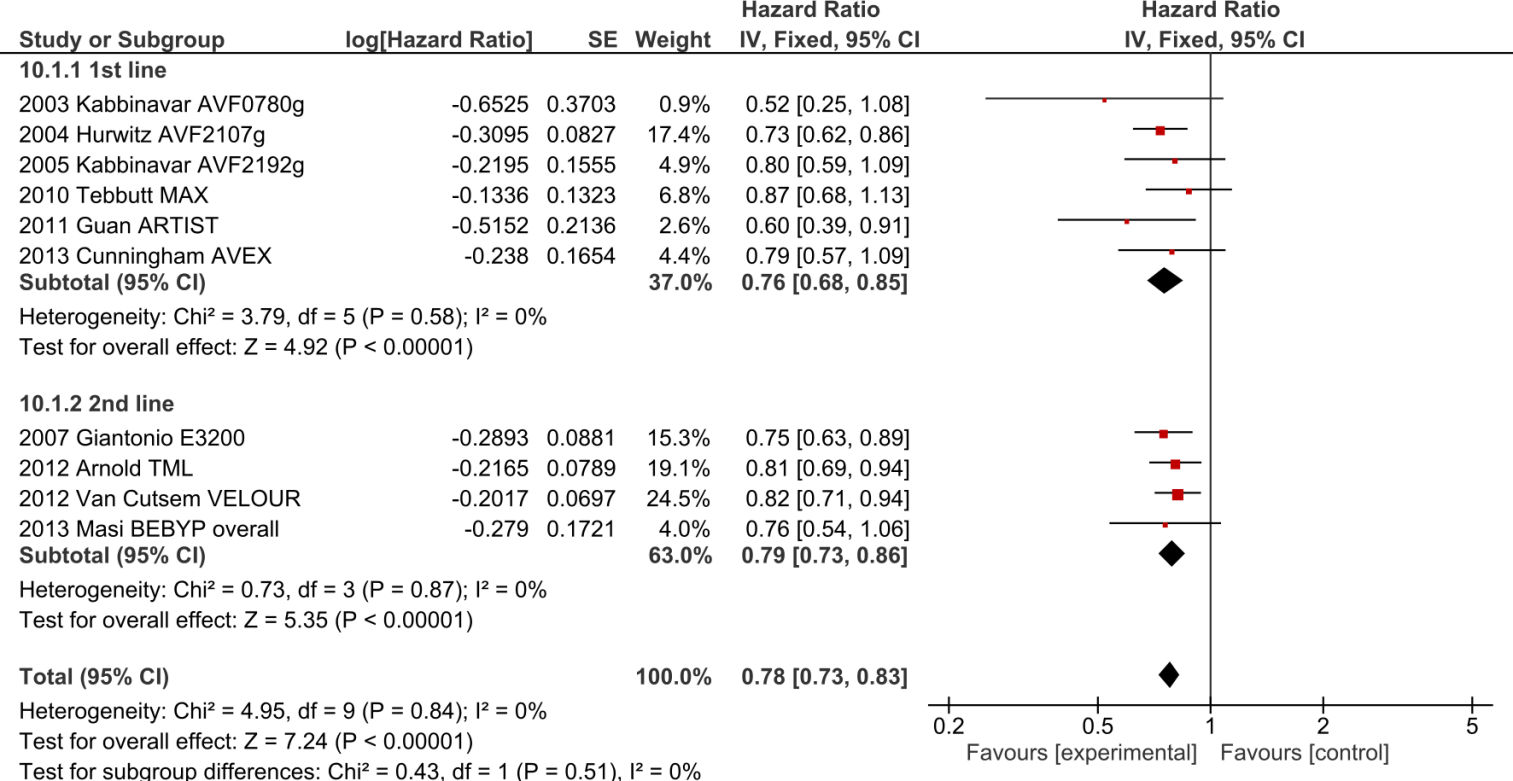
OS outcomes for EGFR-I by chemotherapy backbone—extended RAS analysis.

**1.4 Sensitivity analyses for EGFR-I trials—extended RAS, cetuximab/panitumumab**. Of the above trials, four trials—two using oxaliplatin (OPUS, PRIME)[31, 32] and two using irinotecan (CRYSTAL, Study 181)[33] have reported outcomes according to extended RAS status. The addition of EGFR-I to oxaliplatin-based chemotherapy resulted in no significant improvement to OS (HR 0.81, 95% CI 0.65–1.00, p = 0.05, Fig 5). The addition of EGFR-I to irinotecan-based chemotherapy did improve OS (HR 0.74, 95% CI 0.63–0.89, p = 0.0009). We note, however, that no significant subgroup differences were detected (I2 = 0%, p = 0.56).

With respect to the secondary outcome of PFS, pooled analysis was also performed. The addition of EGFR-I to oxaliplatin-based chemotherapy improved PFS (HR 0.70, 95% CI 0.57–0.86, p = 0.0009, S5 Fig). The addition of EGFR-I to irinotecan-based chemotherapy also improved PFS (HR 0.64, 95% CI 0.52–0.78, p<0.00001). Again, no significant subgroup differences were detected (I2 = 0%, p = 0.52). No significant statistical heterogeneity was present for either of the above analyses.

We conducted additional analyses to determine whether the choice of cetuximab or panitumumab may have influenced the results of our analysis, and found that the results were not affected. When only trials investigating cetuximab were included (4 oxaliplatin, 2 irinotecan), addition of EGFR-I to oxaliplatin-based chemotherapy did not improve OS (HR 1.02, 95% CI 0.88–1.17, p = 0.480, S6 Fig) nor PFS (HR 0.98, 95% CI 0.87–1.11, p = 0.80, S7 Fig). Addition of EGFR-I to irinotecan-based chemotherapy improved OS (HR 0.80, 95% CI 0.67–0.95, p = 0.01) as well as PFS (HR 0.69, 95% CI 0.56–0.86, p = 0.0007). There was again significant subgroup interaction favouring the irinotecan-based arm with regard OS (I2 = 77.9%, p = 0.03) and PFS (I2 = 87.3%, p = 0.005).

Repeating the analysis performed in 1.1.1 (Impact of FP type on Oxaliplatin + EGFR-I) restricted to trials utilizing cetuximab confirmed that there was no significant subgroup interaction in the OS analysis. Moderate subgroup interactions were still present for PFS (I^2^ = 40%, p = 0.19, S8 Fig) favouring infusional 5FU (HR 0.85, 95% CI 0.69–1.05) over bolus 5FU (HR 1.07, 95% CI 0.79–1.45) and capecitabine (HR 1.09, 95% CI 0.91–1.30). Given that only 4 trials were involved overall in this analysis (OPUS, COIN, NEW EPOC, NORDIC VII), this analysis should be interpreted with caution.

With regards panitumumab, given that there was only one oxaliplatin and two irinotecan-based trials, meta-analysis was not performed.

#### 2. The effect of chemotherapy partner on efficacy of anti-angiogenesis agents

**Fig 6:**
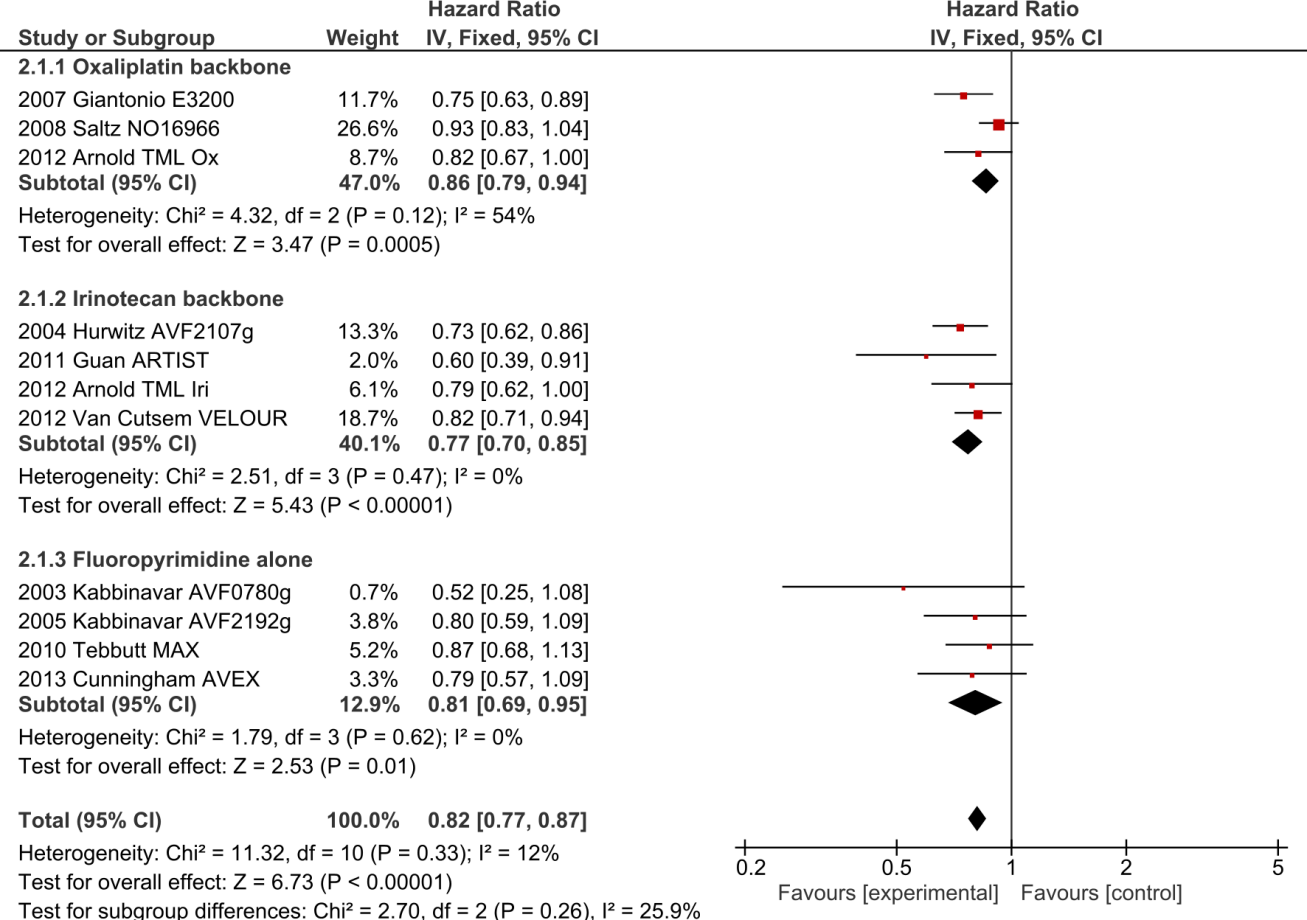
OS outcomes for anti-angiogenic agents by chemotherapy backbone.

**Fig 7:**
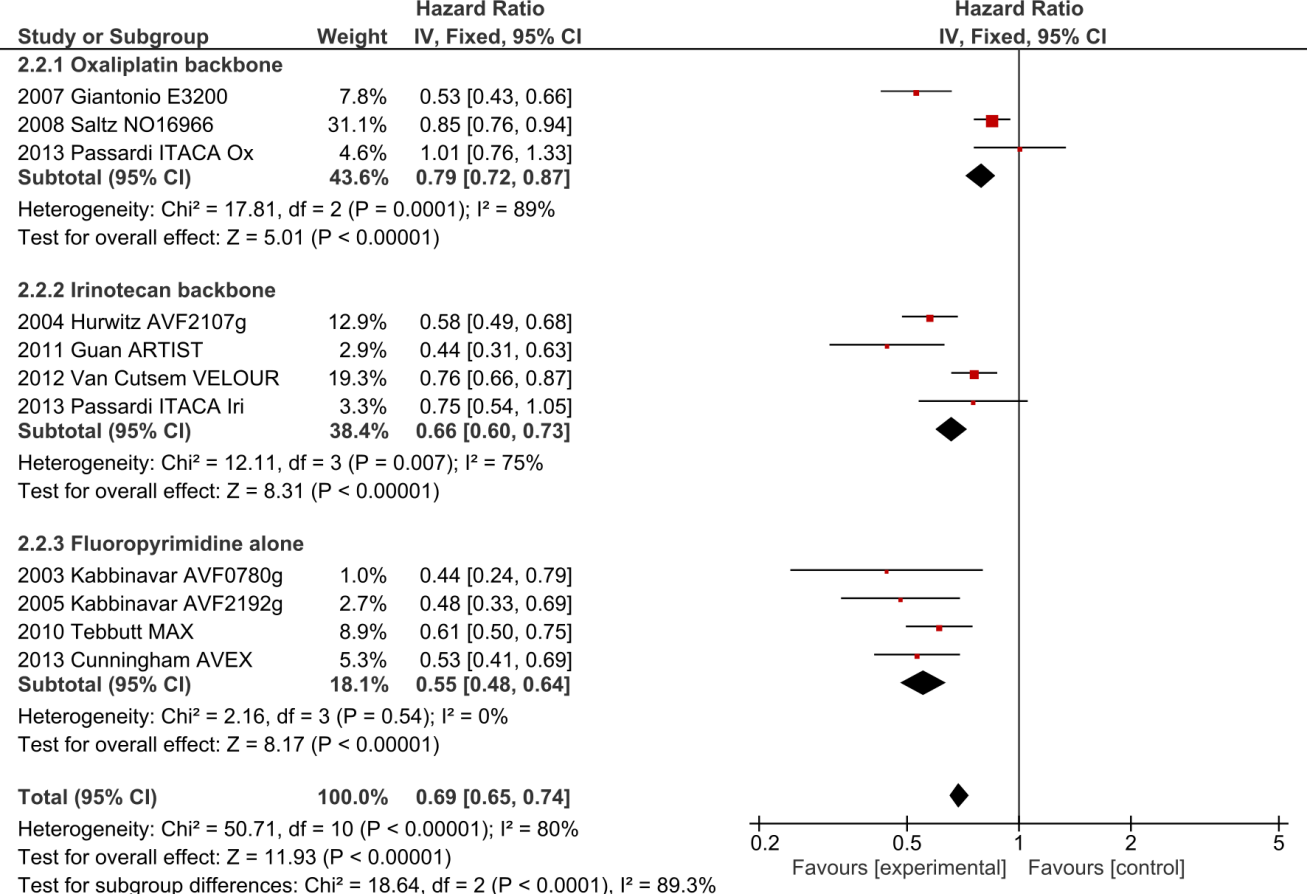
PFS outcomes for anti-angiogenic agents by chemotherapy backbone.

**2.1 Oxaliplatin backbone + bevacizumab**. Four trials (NO16966^15^, E3200^6^, TML^1^ and ITACA[13]) involving 2675 patients investigated the addition of bevacizumab to oxaliplatin-based chemotherapy. No aflibercept trials were reported in sufficient detail for analysis. The addition of bevacizumab significantly improved OS (HR 0.86, 95% CI 0.79–0.94, p = 0.0005, Fig 6) and PFS (HR 0.79, 95% CI 0.72–0.87, p<0.0001, Fig 7). ORR was improved by 4.2% with OR 1.21 (95% CI 1.01–1.46, p = 0.04). Significant heterogeneity was present for OS (I^2^ = 54%), PFS (I^2^ = 89%) and ORR (I^2^ = 88%), possibly due to pooling of bevacizumab studies with differential benefit in different lines of therapy. Random-effects modelling confirmed maintenance of OS benefit, but PFS benefit (HR 0.76, 95% CI 0.55–1.07) and ORR benefit (OR 1.50, 95% CI 0.76–2.97) were no longer significant.

2.1.1. Impact of FP type on oxaliplatin + bevacizumab: Analysis by type of FP was performed in the NO16966 and E3200 studies. TML was excluded as separate results for the multiple types of FP used (XELOX, XELIRI, FOLFOX and FOLFIRI) were not available. No significant subgroup differences by type of FP were present. For OS, HR for the infusional group was 0.77 (95% CI 0.65–0.90), for the capecitabine group 0.78 (95% CI 0.53–1.15) with subgroup interaction values I^2^ = 0%, p = 0.93. For PFS, HR for the infusional group was 0.70 (95% CI 0.60–0.81) and for capecitabine 0.72 (95% CI 0.50–1.04) with subgroup interaction values I^2^ = 0%, p = 0.387.

**2.2. Irinotecan backbone + bevacizumab/aflibercept**. Four bevacizumab trials (AVF2107g[28], ARTIST[7], TML[1] and ITACA[13],) and one aflibercept study (VELOUR [29]), involving 2734 patients, investigated the addition of AIs to irinotecan-based chemotherapy. The addition of AIs improved OS (HR 0.77, 95% CI 0.70–0.85, p<0.0001, Fig 5B) as well as PFS (HR 0.66, 95% CI 0.60–0.73, p<0.00001, Fig 6). ORR was improved by 4.5% with OR 1.30 (95% CI 1.09–1.56, p = 0.004). Significant heterogeneity was present for PFS (I^2^ = 75%, p = 0.007), ORR (I^2^ = 73%, p = 0.02) and toxicity (I^2^ = 72%, p = 0.03), likely due to differences in the chemotherapy backbones and agents (mIFL with bevacizumab in AVF2107g and ARTIST, FOLFIRI + aflibercept in VELOUR). Random-effects modelling confirmed maintenance of PFS benefit but ORR benefit was no longer significant (OR 1.44, 95% CI 0.96–2.16).

2.2.1 Impact of FP type on irinotecan + bevacizumab/aflibercept: Analysis by type of FP was performed in the AVF2107g (mIFL), ARTIST (mIFL), ITACA (FOLFIRI) and VELOUR (FOLFIRI) trials. As in 2.1.1, TML was excluded. For OS, the HR for the infusional group was 0.81 (95% CI 0.72–0.91) and for the bolus group 0.71 (95% CI 0.61–0.83), with subgroup interaction values I^2^ = 40.4%, p = 0.20. For PFS, the HR for the infusional group was 0.76 (95% CI 0.67–0.86) and for the bolus group 0.55 (95% CI 0.47–0.64). Although significant subgroup interaction was noted between infusional and bolus 5FU groups in PFS (I^2^ = 90.3%, p = 0.001), we note that the bulk of the statistical power in the infusional 5FU group (50.3% out of 58.8% weight) was contributed to by the VELOUR study, evaluating aflibercept in the second-line setting.

**2.3. Single agent FP + bevacizumab.** Two trials using infusional 5-Fluorouracil (AVF0780g[8], AVF2192g[9]), and two using capecitabine (MAX[17], AVEX[5, 8, 9, 17]) involving 1064 patients investigated the addition of bevacizumab to single agent FP. The addition of bevacizumab significantly improved OS (HR 0.81, 95% CI 0.69–0.95, p = 0.01,Fig 5C) and PFS (HR 0.55, 95% CI 0.48–0.64, p<0.00001,Fig 6). ORR was improved with pooled ORR increased by 10.1% (OR 1.77 (95% CI 1.28–2.46, p = 0.006)). No significant heterogeneity was present. Analysing by type of FP, no significant subgroup interactions were noted.

**2.4. Interaction between oxaliplatin, irinotecan and single-agent FP with anti-angiogenic agents.** Analysing these three regimens in AI trials, significant subgroup interactions were present with regards to PFS in favour of FP alone (I^2^ = 89.3%, p<0.0001, Fig 6), but no interactions were observed in OS (I^2^ = 25.9%, p = 0.26, Fig 5) or ORR (I^2^ = 49.7%, p = 0.14). The oxaliplatin and irinotecan groups were compared after exclusion of FP-only trials. Oxaliplatin-irinotecan subgroup interaction values were I^2^ = 85.5%, p = 0.009 for PFS and I^2^ = 62.8%, p = 0.10 for OS, suggesting greater benefit from combining irinotecan-based regimens with VEGF inhibitors compared to oxaliplatin-based regimens. Considering infusional 5FU trials only (i.e. bevacizumab with FOLFOX versus with FOLFIRI), the PFS interaction was no longer present (I2 = 0%, p = 0.42).

#### 3. Trials directly comparing different chemotherapy backbones with same targeted agent

Four trials (CELIM, KRK0104, CECOG, Schmeigel 2013[34–36]) evaluating a total of 262 patients investigated combination of biological therapy (cetuximab in 3 studies, bevacizumab in Schmeigel) with different chemotherapy backbones. Limited outcome data were available for the four studies. For the three cetuximab studies, no significant differences were observed for OS (HR 1.20, 95% CI 0.85–1.70), PFS (meta-analysis not performed as only one trial), or ORR (OR 1.25, 95% CI 0.64–2.45). Meta-analysis was not performed for the single bevacizumab study, which showed no significant differences in OS or PFS between CAPOX+B and CAPIRI+B (although it was not specifically powered for these endpoints).

### Sensitivity analysis

We investigated the impact of excluding the NEW EPOC study, which investigated the addition of perioperative cetuximab for resectable liver metastases, as this clinical setting involving curative attempt surgery was distinctly different to the metastatic setting of the other studies. PFS HR was improved somewhat for oxaliplatin regimens with EGFR-I (HR 0.88, 95% CI 0.80–0.98) but unchanged for irinotecan regimens.

Similarly, we explored the exclusion of VELOUR in irinotecan-AI trials (2.2) due to the different mode of action of aflibercept compared to bevacizumab. Benefit was maintained for PFS (HR 0.58, 95% CI 0.50–0.66) and OS (HR 0.73, 95% CI 0.65–0.83).

### Toxicity and quality of life

The addition of biologic agents resulted in increased overall rates of toxicity (S9 and S10 Figs). Only 7/22 trials reported quality of life outcomes using validated tools (S1 Table). The PICCOLO and AVF2192g studies reported improved quality of life in the experimental arm with other trials showing no significant difference.

Considering toxicity outcomes according to chemotherapy partner, no significant subgroup interaction was observed (I^2^ = 60.6%, p = 0.11) for addition of EGFR-I but less toxicity was found adding AIs to oxaliplatin-based trials compared to irinotecan-based trials (I^2^ = 90.1%, p = 0.002).

## Discussion

Whilst biologic agents have improved outcomes for patients with mCRC and are integrated into treatment guidelines, the issue of the optimal combination and sequencing of agents remains unclear. This study is the first to systematically examine the effect of chemotherapy backbone, including fluoropyrimidine choice, on the efficacy of biological treatment in mCRC.

Considering the addition of EGFR-I to chemotherapy in KRAS exon 2 WT patients, benefits in OS, PFS and ORR were found in combination with irinotecan-based but not oxaliplatin-based chemotherapy. Investigating the EGFR-I + oxaliplatin subgroup more closely, superior efficacy was observed in trials utilizing infusional 5FU over those using capecitabine. Subsequent analysis of infusional FP based trials alone demonstrated remarkably similar efficacy between the two backbones, pointing to the use of capecitabine as a possible cause for the lower efficacy of EGFR-I when used in combination with oxaliplatin.

This study expands on the meta-analysis by Vale et al [24] by including data from PICCOLO and NEW EPOC, and confirms that FP choice may be responsible for differential efficacy of adding EGFR-I to ox chemotherapy. We also note the meta-analysis performed by Loupakis et al [37] of anti-EGFR agents in the first line setting. We build upon this by including anti-EGFR trials in all lines, trials investigating anti-angiogenesis agents and perform further subgroup analyses. Given this consistent and independent finding, the available evidence suggests that infusional 5-FU regimens combined with oxaliplatin and EGFR-I may be preferable to bolus 5-FU or capecitabine combinations, notwithstanding other factors affecting choice of regimen such as toxicity and patient preferences.

Two hypotheses may explain the apparent differential activity between type of FP and EGFR-I. One explanation may be increased toxicity from capecitabine-containing regimens with resultant decreased total dose intensity and hence efficacy. Patients in the XELOX arm of the COIN trial received a shorter duration of treatment, median 25.1 weeks in XELOX versus the FOLFOX arm (28.1 weeks). Diarrhoea (23% vs 16% in treatment arms), HFS (16% vs 4%) and stomatitis (4% vs 1%) were all increased in the XELOX arm and may have led the protocol amendment mid-study reducing the dose of capecitabine from 1000 to 850mg/m^2^ bid (which also carried through to the NEW EPOC study).

Another hypothesis, albeit speculative, involves the fact that capecitabine requires metabolic activation within cells to its active form as opposed to 5-FU. Cetuximab leads to G1 arrest and thus decreased cell cycling might lead to less cytotoxic activity.

There is scant information as to whether capecitabine combined with irinotecan has deleterious effects on EGFR-I efficacy; the only trial identified investigating this combination was KRK-0104, directly comparing CAPIRI+C and CAPOX+C (cited above) which showed no significant differences in efficacy.

Recently, retrospective analyses of large EGFR-I trials including PRIME[32], FIRE-3[38], CRYSTAL[33] and OPUS[31] have demonstrated restriction of treatment benefit to extended RAS WT populations (KRAS exons 2, 3 and 4 as well as NRAS exons 2, 3 and 4).

CALGB 80405[39], comparing the use of cetuximab and bevacizumab, showed no OS efficacy difference in both KRAS exon 2 WT and extended RAS WT populations (although higher response rate– 68.6% vs 53.6%, p<0.01 –was achieved with cetuximab in extended RAS WT populations).

The combination of the AIs bevacizumab and aflibercept with chemotherapy improved OS, PFS and ORR with benefit preserved across both oxaliplatin- and irinotecan-based backbones. Subgroup interaction testing favoured increased efficacy for irinotecan. This finding was reported previously but in a pooled analysis of 3763 patients only[26]. This systematic review confirms these findings and also includes additional trials (VELOUR and AVEX).

Restriction to trials of AIs using infusional-only FP in combination with either ox or iri showed a persistent significant PFS benefit but no further subgroup interaction. This interaction is difficult to interpret given the VELOUR contributed to the bulk of the statistical power in the FOLFIRI analysis. A Phase II RCT with FOLFOX+aflibercept has been incompletely reported[14] and we were unable to include it in the analysis. Whilst there was evidence for increased efficacy of bevacizumab added to single-agent FP compared to FP chemotherapy alone, the lesser activity of single-agent FP means that it is usually reserved for elderly or frail patients in routine clinical practice.

A separate question not explicitly addressed by the study is which biological agent optimally combines with which chemotherapy agent (i.e. chemo + EGFR-I first then chemo + AI or vice versa). Whilst FIRE-3 and PEAK point to the possibly increased efficacy of EGFR-I in RAS WT patients, their restriction to one chemotherapy regimen (FOLFIRI in FIRE-3, FOLFOX in PEAK) mean that they cannot definitively answer the questions posed by this paper about chemotherapy backbone choice. We note other studies recently published that address this question. [40]

The strengths of this study include the systematic review of all relevant trials and the rigorous methodology. The large number of patients included in analysis helps draw top-level conclusions about the subject matter. The suggestion that FP choice may be responsible for negative interactions between oxaliplatin-based chemotherapies and EGFR-I provides scope for further research.

We recognize several limitations to this study, including restriction of analysis to publication-only results, statistical heterogeneity and the relatively small number of patients in direct comparison trials.

The above meta-analysis has several implications for practice in mCRC. Assuming the availability of all agents, it would seem best to combine EGFR-I with FOLFIRI or FOLFOX based regimens. Based on the available data, CAPOX partnered with EGFR-I appears to be the least effective.

In contrast to the above, AIs may be combined with either oxaliplatin-based or irinotecan-based options. The improved efficacy of AIs added to fluoropyrimidine monotherapy may reflect their greater effectiveness in less active regimens. This points to the importance of considering use of targeted agents even in frailer patients.

Whilst this study raises interesting possibilities of an interaction between cetuximab, oxaliplatin and capecitabine, the biological basis underlying the combination of agents has not been fully elucidated and this study points to the importance of ongoing research in this area.

## Conclusions

EGFR-I are best used in combination with irinotecan based regimens or with infusional FP regimens when combined with oxaliplatin. Capecitabine-oxaliplatin combinations with EGFR-I appear less effective. No statistically significant difference in efficacy is seen when AIs are used with both irinotecan or oxaliplatin based regimens.

## Supporting Information

S1 Fig CONSORT diagram. (TIF)

S2 Fig Funnel plot for anti-angiogenic agents—PFS. (TIF)

S3 Fig OS outcomes for oxaliplatin + EGFR-I by FP backbone. (TIF)

S4 Fig PFS outcomes for EGFR-I—restricted to infusional-only populations. (TIF)

S5 Fig PFS outcomes for EGFR-I by chemotherapy backbone—extended RAS analysis. (TIF)

S6 Fig OS outcomes for EGFR-I by chemotherapy backbone—cetuximab only. (TIF)

S7 Fig PFS outcomes for EGFR-I by chemotherapy backbone—cetuximab only. (TIF)

S8 Fig PFS outcomes for oxaliplatin + EGFR-I by FP backbone—restricted to cetuximab trials only. (TIF)

S9 Fig Overall Grade 3/4 Toxicity outcomes for EGFR-Is. (TIF)

S10 Fig Overall Grade 3/4 Toxicity outcomes for AIs. (TIF)

S1 Methods Sample search strategy. (DOCX)

S1 PRISMA Checklist PRISMA checklist. (DOC)

S1 Table Quality of life outcomes for included trials. (DOC)

## References

[1] Bennouna J, Sastre J, Arnold D, Osterlund P, Greil R, Van Cutsem E, et al Continuation of bevacizumab after first progression in metastatic colorectal cancer (ML18147): a randomised phase 3 trial. The lancet oncology. 2013;14(1):29–37. 10.1016/S1470-2045(12)70477-1 . 23168366

[2] Bokemeyer C, Bondarenko I, Makhson A, Hartmann JT, Aparicio J, de Braud F, et al Fluorouracil, leucovorin, and oxaliplatin with and without cetuximab in the first-line treatment of metastatic colorectal cancer. Journal of clinical oncology: official journal of the American Society of Clinical Oncology. 2009;27(5):663–71. 10.1200/JCO.2008.20.8397 . 19114683

[3] Borner M, Koeberle D, Von Moos R, Saletti P, Rauch D, Hess V, et al Adding cetuximab to capecitabine plus oxaliplatin (XELOX) in first-line treatment of metastatic colorectal cancer: a randomized phase II trial of the Swiss Group for Clinical Cancer Research SAKK. Annals of oncology: official journal of the European Society for Medical Oncology / ESMO. 2008;19(7):1288–92. 10.1093/annonc/mdn058 . 18349029

[4] Cassidy J, Clarke S, Diaz-Rubio E, Scheithauer W, Figer A, Wong R, et al XELOX vs FOLFOX-4 as first-line therapy for metastatic colorectal cancer: NO16966 updated results. British journal of cancer. 2011;105(1):58–64. 10.1038/bjc.2011.201 21673685 PMC3137415

[5] Cunningham D, Lang I, Marcuello E, Lorusso V, Ocvirk J, Shin DB, et al Bevacizumab plus capecitabine versus capecitabine alone in elderly patients with previously untreated metastatic colorectal cancer (AVEX): an open-label, randomised phase 3 trial. The lancet oncology. 2013;14(11):1077–85. 10.1016/S1470-2045(13)70154-2 . 24028813

[6] Giantonio BJ, Catalano PJ, Meropol NJ, O'Dwyer PJ, Mitchell EP, Alberts SR, et al Bevacizumab in combination with oxaliplatin, fluorouracil, and leucovorin (FOLFOX4) for previously treated metastatic colorectal cancer: results from the Eastern Cooperative Oncology Group Study E3200. Journal of clinical oncology: official journal of the American Society of Clinical Oncology. 2007;25(12):1539–44. 10.1200/JCO.2006.09.6305 . 17442997

[7] Guan ZZ, Xu JM, Luo RC, Feng FY, Wang LW, Shen L, et al Efficacy and safety of bevacizumab plus chemotherapy in Chinese patients with metastatic colorectal cancer: a randomized phase III ARTIST trial. Chinese journal of cancer. 2011;30(10):682–9. 10.5732/cjc.011.10188 . 21959045 PMC4012268

[8] Kabbinavar F, Hurwitz HI, Fehrenbacher L, Meropol NJ, Novotny WF, Lieberman G, et al Phase II, randomized trial comparing bevacizumab plus fluorouracil (FU)/leucovorin (LV) with FU/LV alone in patients with metastatic colorectal cancer. Journal of clinical oncology: official journal of the American Society of Clinical Oncology. 2003;21(1):60–5. . 12506171 10.1200/JCO.2003.10.066

[9] Kabbinavar FF, Schulz J, McCleod M, Patel T, Hamm JT, Hecht JR, et al Addition of bevacizumab to bolus fluorouracil and leucovorin in first-line metastatic colorectal cancer: results of a randomized phase II trial. Journal of clinical oncology: official journal of the American Society of Clinical Oncology. 2005;23(16):3697–705. 10.1200/JCO.2005.05.112 . 15738537

[10] Kabbinavar FF, Wallace JF, Holmgren E, Yi J, Cella D, Yost KJ, et al Health-related quality of life impact of bevacizumab when combined with irinotecan, 5-fluorouracil, and leucovorin or 5-fluorouracil and leucovorin for metastatic colorectal cancer. The oncologist. 2008;13(9):1021–9. 10.1634/theoncologist.2008-0003 . 18776057

[11] Masi G, L F, Salvatore L., et al Second-line chemotherapy (CT) with or without bevacizumab (BV) in metastatic colorectal cancer (mCRC) patients (pts) who progressed to a first-line treatment containing BV: Updated results of the phase III “BEBYP” trial by the Gruppo Oncologico Nord Ovest (GONO). Journal of clinical oncology: official journal of the American Society of Clinical Oncology. 2013;31((suppl; abstr 3615)).

[12] Maughan TS, Adams RA, Smith CG, Meade AM, Seymour MT, Wilson RH, et al Addition of cetuximab to oxaliplatin-based first-line combination chemotherapy for treatment of advanced colorectal cancer: results of the randomised phase 3 MRC COIN trial. Lancet. 2011;377(9783):2103–14. 10.1016/S0140-6736(11)60613-2 21641636 PMC3159415

[13] Passardi A, Nanni O, Tassinari D, Turci D, Cavanna L, Fontana A, et al Effectiveness of bevacizumab added to standard chemotherapy in metastatic colorectal cancer: final results for first-line treatment from the ITACa randomized clinical trial. Annals of oncology: official journal of the European Society for Medical Oncology/ESMO. 2015. 10.1093/annonc/mdv130 25735317

[14] Pericay CFG, Saunders M., Thomas A., Roh J., Lopez R., et al Phase 2 randomized, noncomparative open-label study of aflibercept and modified FOLFOX6 in the first line treatment of metastatic colorectal cancer (AFFIRM). Ann Oncol 2012;23((Suppl. 4): iv16, abstract 0024.).

[15] Saltz LB, Clarke S, Diaz-Rubio E, Scheithauer W, Figer A, Wong R, et al Bevacizumab in combination with oxaliplatin-based chemotherapy as first-line therapy in metastatic colorectal cancer: a randomized phase III study. Journal of clinical oncology: official journal of the American Society of Clinical Oncology. 2008;26(12):2013–9. 10.1200/JCO.2007.14.9930 . 18421054

[16] Seymour MT, Brown SR, Middleton G, Maughan T, Richman S, Gwyther S, et al Panitumumab and irinotecan versus irinotecan alone for patients with KRAS wild-type, fluorouracil-resistant advanced colorectal cancer (PICCOLO): a prospectively stratified randomised trial. The lancet oncology. 2013;14(8):749–59. 10.1016/S1470-2045(13)70163-3 23725851 PMC3699713

[17] Tebbutt NC, Wilson K, Gebski VJ, Cummins MM, Zannino D, van Hazel GA, et al Capecitabine, bevacizumab, and mitomycin in first-line treatment of metastatic colorectal cancer: results of the Australasian Gastrointestinal Trials Group Randomized Phase III MAX Study. Journal of clinical oncology: official journal of the American Society of Clinical Oncology. 2010;28(19):3191–8. 10.1200/JCO.2009.27.7723 . 20516443

[18] Tveit KM, Guren T, Glimelius B, Pfeiffer P, Sorbye H, Pyrhonen S, et al Phase III trial of cetuximab with continuous or intermittent fluorouracil, leucovorin, and oxaliplatin (Nordic FLOX) versus FLOX alone in first-line treatment of metastatic colorectal cancer: the NORDIC-VII study. Journal of clinical oncology: official journal of the American Society of Clinical Oncology. 2012;30(15):1755–62. 10.1200/jco.2011.38.0915 . 22473155

[19] Van Cutsem E, Kohne CH, Hitre E, Zaluski J, Chang Chien CR, Makhson A, et al Cetuximab and chemotherapy as initial treatment for metastatic colorectal cancer. The New England journal of medicine. 2009;360(14):1408–17. 10.1056/NEJMoa0805019 . 19339720

[20] Ye LC, Liu TS, Ren L, Wei Y, Zhu DX, Zai SY, et al Randomized controlled trial of cetuximab plus chemotherapy for patients with KRAS wild-type unresectable colorectal liver-limited metastases. Journal of clinical oncology: official journal of the American Society of Clinical Oncology. 2013;31(16):1931–8. 10.1200/JCO.2012.44.8308 . 23569301

[21] Douillard JY, Siena S, Cassidy J, Tabernero J, Burkes R, Barugel M, et al Randomized, phase III trial of panitumumab with infusional fluorouracil, leucovorin, and oxaliplatin (FOLFOX4) versus FOLFOX4 alone as first-line treatment in patients with previously untreated metastatic colorectal cancer: the PRIME study. Journal of clinical oncology: official journal of the American Society of Clinical Oncology. 2010;28(31):4697–705. 10.1200/JCO.2009.27.4860 . 20921465

[22] Amado RG, Wolf M, Peeters M, Van Cutsem E, Siena S, Freeman DJ, et al Wild-type KRAS is required for panitumumab efficacy in patients with metastatic colorectal cancer. Journal of clinical oncology: official journal of the American Society of Clinical Oncology. 2008;26(10):1626–34. 10.1200/JCO.2007.14.7116 . 18316791

[23] Karapetis CS, Khambata-Ford S, Jonker DJ, O'Callaghan CJ, Tu D, Tebbutt NC, et al K-ras mutations and benefit from cetuximab in advanced colorectal cancer. The New England journal of medicine. 2008;359(17):1757–65. 10.1056/NEJMoa0804385 . 18946061

[24] Vale CL, Tierney JF, Fisher D, Adams RA, Kaplan R, Maughan TS, et al Does anti-EGFR therapy improve outcome in advanced colorectal cancer? A systematic review and meta-analysis. Cancer treatment reviews. 2012;38(6):618–25. 10.1016/j.ctrv.2011.11.002 . 22118887

[25] Zhou SW, Huang YY, Wei Y, Jiang ZM, Zhang YD, Yang Q, et al No survival benefit from adding cetuximab or panitumumab to oxaliplatin-based chemotherapy in the first-line treatment of metastatic colorectal cancer in KRAS wild type patients: a meta-analysis. PloS one. 2012;7(11):e50925 10.1371/journal.pone.0050925 23226426 PMC3511401

[26] Hurwitz HI, Tebbutt NC, Kabbinavar F, Giantonio BJ, Guan ZZ, Mitchell L, et al Efficacy and safety of bevacizumab in metastatic colorectal cancer: pooled analysis from seven randomized controlled trials. The oncologist. 2013;18(9):1004–12. 10.1634/theoncologist.2013-0107 23881988 PMC3780632

[27] Primrose J, Falk S, Finch-Jones M, Valle J, O'Reilly D, Siriwardena A, et al Systemic chemotherapy with or without cetuximab in patients with resectable colorectal liver metastasis: the New EPOC randomised controlled trial. The lancet oncology. 2014;15(6):601–11. 10.1016/S1470-2045(14)70105-6 24717919

[28] Hurwitz H, Fehrenbacher L, Novotny W, Cartwright T, Hainsworth J, Heim W, et al Bevacizumab plus irinotecan, fluorouracil, and leucovorin for metastatic colorectal cancer. The New England journal of medicine. 2004;350(23):2335–42. 10.1056/NEJMoa032691 . 15175435

[29] Van Cutsem E, Tabernero J, Lakomy R, Prenen H, Prausova J, Macarulla T, et al Addition of aflibercept to fluorouracil, leucovorin, and irinotecan improves survival in a phase III randomized trial in patients with metastatic colorectal cancer previously treated with an oxaliplatin-based regimen. Journal of clinical oncology: official journal of the American Society of Clinical Oncology. 2012;30(28):3499–506. 10.1200/JCO.2012.42.8201 . 22949147

[30] Sobrero AF, Maurel J, Fehrenbacher L, Scheithauer W, Abubakr YA, Lutz MP, et al EPIC: phase III trial of cetuximab plus irinotecan after fluoropyrimidine and oxaliplatin failure in patients with metastatic colorectal cancer. Journal of clinical oncology: official journal of the American Society of Clinical Oncology. 2008;26(14):2311–9. 10.1200/JCO.2007.13.1193 . 18390971

[31] Bokemeyer C, Kohne CH, Ciardiello F, Lenz HJ, Heinemann V, Klinkhardt U, et al FOLFOX4 plus cetuximab treatment and RAS mutations in colorectal cancer. European journal of cancer. 2015;51(10):1243–52. 10.1016/j.ejca.2015.04.007 . 25937522 PMC7508202

[32] Douillard J-Y, Oliner KS, Siena S, Tabernero J, Burkes R, Barugel M, et al Panitumumab–FOLFOX4 treatment and RAS mutations in colorectal cancer. New England Journal of Medicine. 2013;369(11):1023–34. 10.1056/NEJMoa1305275 24024839

[33] Van Cutsem E, Lenz H-J, Köhne C-H, Heinemann V, Tejpar S, Melezínek I, et al Fluorouracil, leucovorin, and irinotecan plus cetuximab treatment and RAS mutations in colorectal cancer. Journal of Clinical Oncology. 2015;33(7):692–700. 10.1200/JCO.2014.59.4812 25605843

[34] Folprecht G, Gruenberger T, Bechstein WO, Raab HR, Lordick F, Hartmann JT, et al Tumour response and secondary resectability of colorectal liver metastases following neoadjuvant chemotherapy with cetuximab: the CELIM randomised phase 2 trial. The lancet oncology. 2010;11(1):38–47. 10.1016/S1470-2045(09)70330-4 . 19942479

[35] Moosmann N, von Weikersthal LF, Vehling-Kaiser U, Stauch M, Hass HG, Dietzfelbinger H, et al Cetuximab plus capecitabine and irinotecan compared with cetuximab plus capecitabine and oxaliplatin as first-line treatment for patients with metastatic colorectal cancer: AIO KRK-0104—a randomized trial of the German AIO CRC study group. Journal of clinical oncology: official journal of the American Society of Clinical Oncology. 2011;29(8):1050–8. 10.1200/JCO.2010.31.1936 . 21300933

[36] Ocvirk J, Brodowicz T, Wrba F, Ciuleanu TE, Kurteva G, Beslija S, et al Cetuximab plus FOLFOX6 or FOLFIRI in metastatic colorectal cancer: CECOG trial. World journal of gastroenterology: WJG. 2010;16(25):3133–43. 20593498 10.3748/wjg.v16.i25.3133 PMC2896750

[37] Loupakis F, Cremolini C, Salvatore L, Schirripa M, Lonardi S, Vaccaro V, et al Clinical impact of anti-epidermal growth factor receptor monoclonal antibodies in first-line treatment of metastatic colorectal cancer: meta-analytical estimation and implications for therapeutic strategies. Cancer. 2012;118(6):1523–32. 10.1002/cncr.26460 . 22009364

[38] Heinemann V, von Weikersthal LF, Decker T, Kiani A, Vehling-Kaiser U, Al-Batran S-E, et al FOLFIRI plus cetuximab versus FOLFIRI plus bevacizumab as first-line treatment for patients with metastatic colorectal cancer (FIRE-3): a randomised, open-label, phase 3 trial. The lancet oncology. 2014;15(10):1065–75. 10.1016/S1470-2045(14)70330-4 25088940

[39] Venook AP, Niedzwiecki D, Lenz HJ, Innocenti F, Mahoney MR, O'Neil BH, et al CALGB/SWOG 80405: Phase III trial of irinotecan/5-FU/leucovorin (FOLFIRI) or oxaliplatin/5-FU/leucovorin (mFOLFOX6) with bevacizumab (BV) or cetuximab (CET) for patients (pts) with KRAS wild-type (wt) untreated metastatic adenocarcinoma of the colon or rectum (MCRC). Journal of clinical oncology: official journal of the American Society of Clinical Oncology. 2014;32(5s):(suppl; abstr LBA3).

[40] Khattak MA, Martin H, Davidson A, Phillips M. Role of First-Line Anti-Epidermal Growth Factor Receptor Therapy Compared With Anti-Vascular Endothelial Growth Factor Therapy in Advanced Colorectal Cancer: A Meta-Analysis of Randomized Clinical Trials. Clinical colorectal cancer. 2015 10.1016/j.clcc.2014.12.011 . 25666296

