# Correction: Does the Chemotherapy Backbone Impact on the Efficacy of Targeted Agents in Metastatic Colorectal Cancer? A Systematic Review and Meta-Analysis of the Literature

**DOI:** 10.1371/journal.pone.0138916

**Published:** 2015-09-25

**Authors:** David L. Chan, Nick Pavlakis, Jeremy Shapiro, Timothy J. Price, Christos S. Karapetis, Niall C. Tebbutt, Eva Segelov

There is an error in reference 22. The correct reference is: Peeters M, Price TJ, Cervantes A, Sobrero AF, Ducreux M, Hotko Y, et al. Randomized phase III study of panitumumab with fluorouracil, leucovorin, and irinotecan(FOLFIRI) compared with FOLFIRI alone as second-line treatment in patients with metastaticcolorectal cancer. J Clin Oncol. 2010 Nov 1;28(31):4706-13.

Fig 5 is incorrect. The authors have provided a corrected version here.

**Fig 5:**
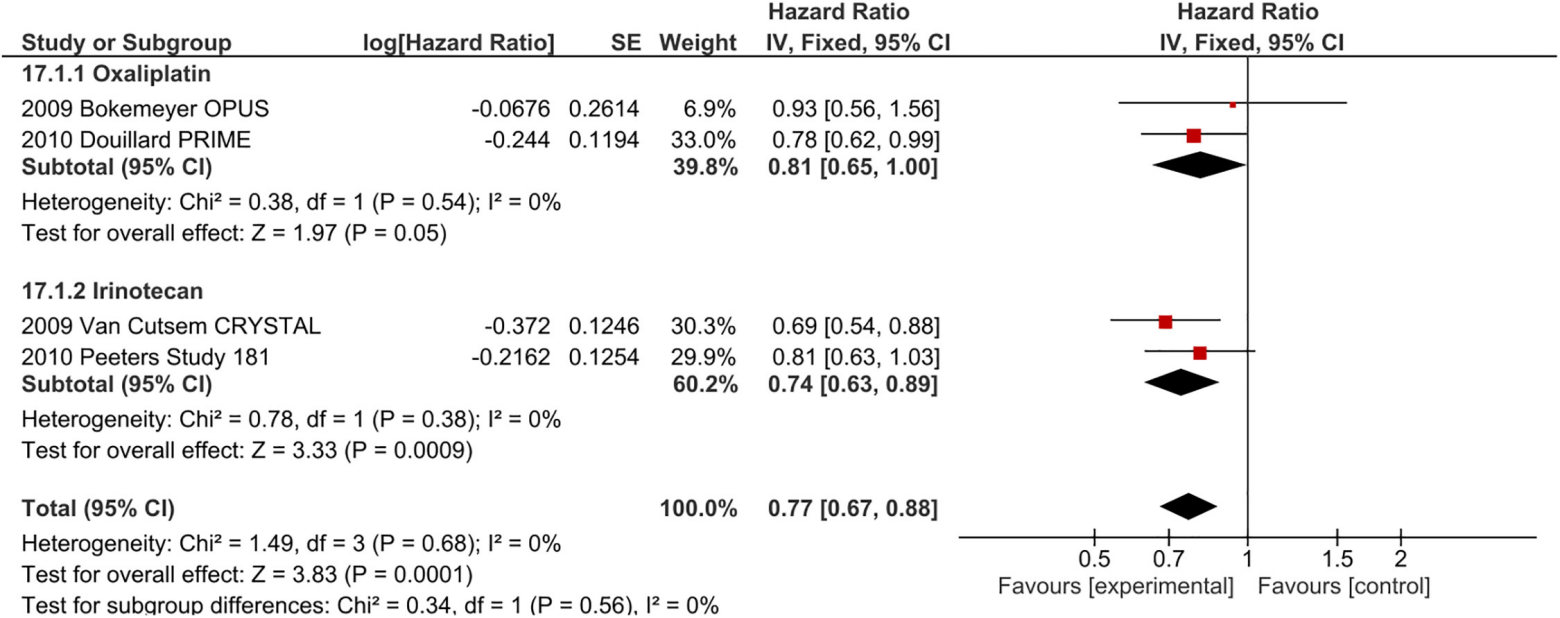
OS outcomes for EGFR-I by chemotherapy backbone—extended RAS analysis.
