# Supplementary figures and images for: Does the Chemotherapy Backbone Impact on the Efficacy of Targeted Agents in Metastatic Colorectal Cancer? A Systematic Review and Meta-Analysis of the Literature
Source: PLoS One. 2015 Aug 14;10(8):e0135599. doi: 10.1371/journal.pone.0135599 (PMC4537274; doi:10.1371/journal.pone.0135599)

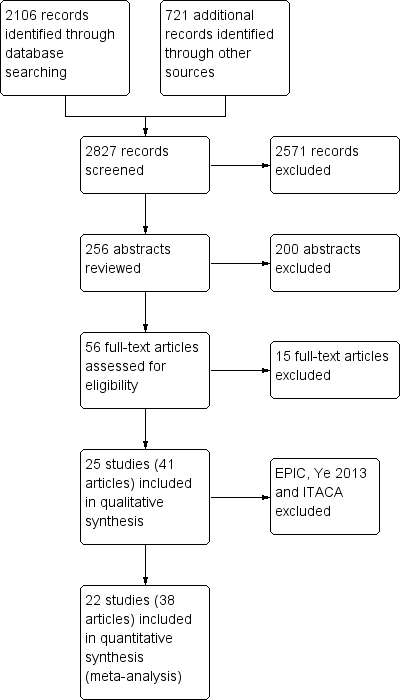

Supplement: S1 Fig — (TIF) [file pone.0135599.s001.tif]

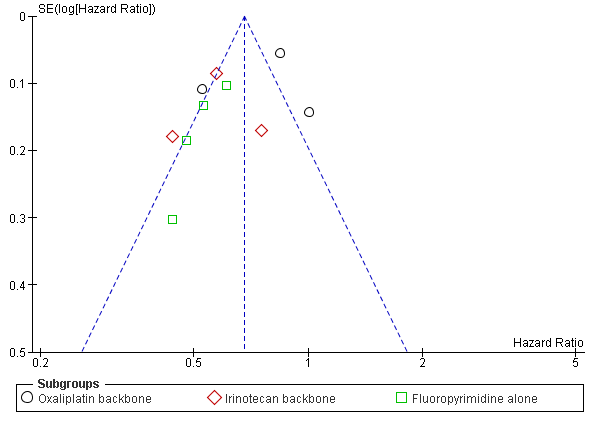

Supplement: S2 Fig — (TIF) [file pone.0135599.s002.tif]

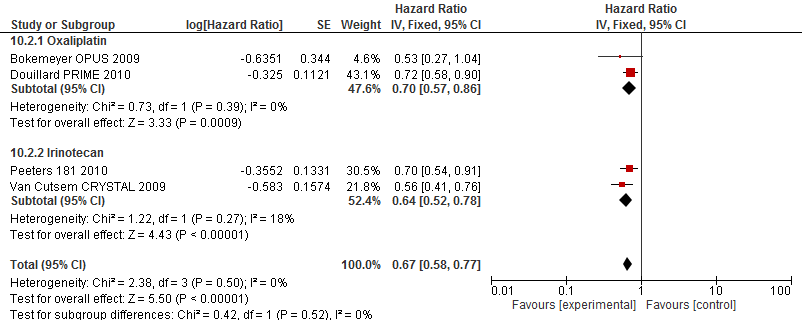

Supplement: S5 Fig — (TIF) [file pone.0135599.s005.tif]

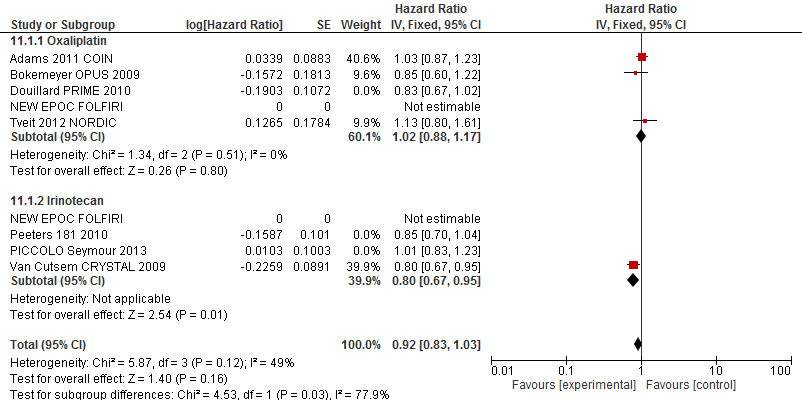

Supplement: S6 Fig — (TIF) [file pone.0135599.s006.tif]

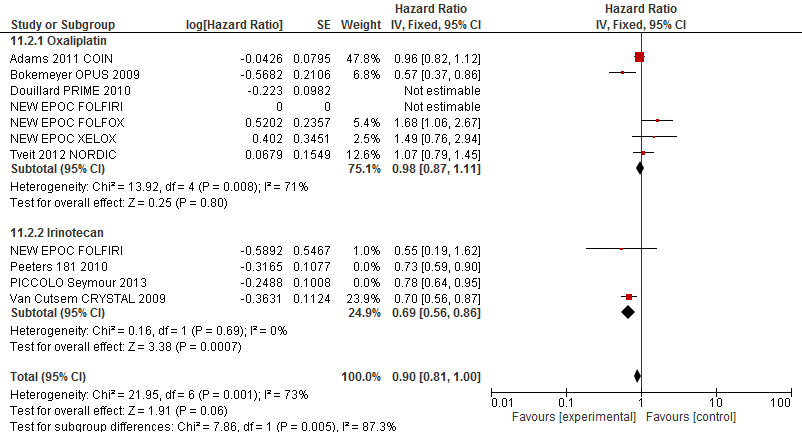

Supplement: S7 Fig — (TIF) [file pone.0135599.s007.tif]

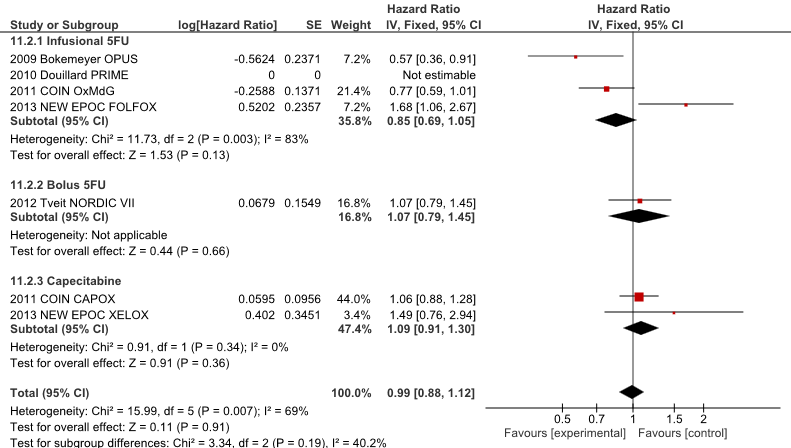

Supplement: S8 Fig — (TIF) [file pone.0135599.s008.tif]
