# Supplementary material for: Does the Chemotherapy Backbone Impact on the Efficacy of Targeted Agents in Metastatic Colorectal Cancer? A Systematic Review and Meta-Analysis of the Literature
Source: PLoS One. 2015 Aug 14;10(8):e0135599. doi: 10.1371/journal.pone.0135599 (PMC4537274; doi:10.1371/journal.pone.0135599)
Supplement: S1 Methods — (DOCX) [file pone.0135599.s011.docx]

***MEDLINE***

1. ((colorect* or colon* or rect* or anal* or anus* or intestin* or bowel*) adj3 (carcinom* or neoplas* or adenocarcinom* or cancer* or tumor* or tumour* or sarcom*)).mp.

2. exp Colorectal Neoplasms/

3. 1 or 2

4. (vascular endothelial growth factor or VEGF or VEGFR or Bevacizumab or Aflibercept or Regorafenib or Brivanib or Pazopanib).mp.

5. exp Vascular Endothelial Growth Factors/

6. exp Receptors, Vascular Endothelial Growth Factor/

7. 4 or 5 or 6

8. exp Neoplasm Metastasis/

9. (metasta* or stage 4 or stage IV or advanced).mp.

10. 8 or 9

11. 3 and 7 and 10

12. randomized controlled trial.pt.

13. controlled clinical trial.pt.

14. randomized.ab.

15. placebo.ab.

16. clinical trial.sh.

17. randomly.ab.

18. trial.ti.

19. 12 or 13 or 14 or 15 or 16 or 17 or 18

20. humans.sh.

21. 19 and 20

22. 11 and 21
