# Supplementary material for: Does the Chemotherapy Backbone Impact on the Efficacy of Targeted Agents in Metastatic Colorectal Cancer? A Systematic Review and Meta-Analysis of the Literature
Source: PLoS One. 2015 Aug 14;10(8):e0135599. doi: 10.1371/journal.pone.0135599 (PMC4537274; doi:10.1371/journal.pone.0135599)
Supplement: S1 Table — (DOC) [file pone.0135599.s013.doc]

| Study title | Treatment | Control | N | QoL instrument | QoL effect | p value | Details |
| --- | --- | --- | --- | --- | --- | --- | --- |
| CRYSTAL [^39^](#_ENREF_39) | FOLFIRI + cetuximab | FOLFIRI | 666 (KRAS WT) | EORTC QLQ-C30: Global and social functioning | No significant difference | 0.12, 0.43 |  |
| COIN | FOLFOX/XELOX + cetuximab | FOLFOX/XELOX | 729 (KRAS WT) | Substudy in progress |  |  |  |
| PICCOLO | Irinotecan + Panitumumab (IrPan) | Irinotecan | 597 | EORTC QLQ-C30, EQ-5D, | Significantly better | 0.032 | QLQ-C30 global scores favoured IrPan group (56.4 vs. 49.5), but QoL symptom scores worse with IrPan |
|  |  |  |  | Dermatology Life Quality Index | No data | N/A |  |
| PRIME[^40^](#_ENREF_40) | FOLFOX + panitumumab | FOLFOX | 576 (KRAS WT) | EQ-5D HIS/VAS | No significant difference | N/A | EQ-5D VAS change 95% CI (-2.925, 1.618) |
| Study 181 [^40^](#_ENREF_40) | FOLFIRI + Panitumumab | FOLFIRI | 530 (KRAS WT) | EQ-5D HIS/VAS | No significant difference | N/A | EQ-5D VAS change 95% CI (-3.280, 0.749) |
| AGITG MAX | Capecitabine + Bevacizumab | Capecitabine | 313 (excluding CBM group) | Questionnaire | No significant difference overall | p>0.1 | Detailed reports to follow |
| AVF2107g[^10^](#_ENREF_10) | mIFL + bevacizumab | mIFL | 249/813 | Time to deterioration in FACT-C total, FACT-C CCS, TOI-C | No significant difference |  | P=.6190 (FACT-C CCS), P=.4618 (TOI-C), p=0.8116 (FACT-C total) |
| AVF2192g[^10^](#_ENREF_10) | 5FU + bevacizumab | 5FU | 166/209 | Time to deterioration in FACT-C total, FACT-C CCS, TOI-C | Improved time to deterioration of FACT-C total | P=0.0396 | P=.2176 (FACT-C CCS), p=0.0537 (TOI-C) |
